# One-Step Multiplex RT-PCR Method for Detection of Melon Viruses

**DOI:** 10.3390/microorganisms12112337

**Published:** 2024-11-15

**Authors:** Sheng Han, Tingting Zhou, Fengqin Zhang, Jing Feng, Chenggui Han, Yushanjiang Maimaiti

**Affiliations:** 1 State Key Laboratory for Agro-Biotechnology, Ministry of Agriculture and Rural Affairs Key Laboratory of Pest Monitoring and Green Management, College of Plant Protection, China Agricultural University, Beijing 100193, China; hanshen_1981@163.com; 2 Key Laboratory of Integrated Pest Management on Crops in Northwestern Oasis, Ministry of Agriculture and Rural Affairs, Institute of Plant Protection, Xinjiang Academy of Agricultural Sciences, Urumqi 830091, China; zhoutingting@xaas.ac.cn (T.Z.); zgyczfq@163.com (F.Z.); 18754801808@163.com (J.F.)

**Keywords:** mixed infection, multiplex detection, viruses, Xinjiang

## Abstract

This study presents a one-step multiplex reverse transcription polymerase chain reaction (RT-PCR) method for the simultaneous detection of multiple viruses affecting melon crops. Viruses such as *Watermelon mosaic virus* (WMV), *Cucumber mosaic virus* (CMV), *Zucchini yellow mosaic virus* (ZYMV), *Squash mosaic virus* (SqMV), *Tobacco mosaic virus* (TMV), *Papaya ring spot virus* (PRSV), and *Melon yellow spot virus* (MYSV) pose a great threat to melons. The mixed infection of these viruses is the most common observation in the melon-growing fields. In this study, we surveyed northern Xingjiang (Altay, Changji, Wujiaqu, Urumqi, Turpan, and Hami) and southern Xingjiang (Aksu, Bayingolin, Kashgar, and Hotan) locations in Xinjiang province and developed a one-step multiplex RT-PCR to detect these melon viruses. The detection limits of this multiplex PCR were 10^3^ copies/μL for ZYMV and MYSV and 10^2^ copies/μL for WMV, SqMV, PRSV, CMV, and TMV. The detection results in the field showed 242 samples were infected by one or more viruses. The multiplex RT-PCR protocol demonstrated rapid, simultaneous, and relatively effective detection of viruses such as WMV, CMV, ZYMV, SqMV, TMV, PRSV, and MYSV. The technique is designed to identify these melon viruses in a single reaction, enhancing diagnostic efficiency and reducing costs, thus serving as a reference for muskmelon anti-virus breeding in Xinjiang.

## 1. Introduction

Plant viruses cause serious diseases to melon [1], watermelon [2], cucumber [3], pumpkin [4], and other important fruit and vegetable crops [2,3]. More than 13 plant viruses have been found and isolated from melon so far [5,6]. These viruses are mainly distributed worldwide and also cause great economic loss in melon-producing areas of China, America, France, and Japan [6,7,8]. In 1980, the incidence of these viruses was more than 90% in some parts of China, and they have been the most serious threat to melon production [9,10]. The co-infection among these viruses has been widely reported in the field [3], compounding the difficulties associated with controlling these viral diseases.

Xinjiang province, known for melon production worldwide, has an extensive cultivation history of Hami and Jiashi melons [9,11]. *Watermelon mosaic virus* (WMV), *Cucumber mosaic virus* (CMV), *Zucchini yellow mosaic virus* (ZYMV), *Squash mosaic virus* (SqMV), *Tobacco mosaic virus* (TMV), *Papaya ring spot virus* (PRSV), and *Melon yellow spot virus* (MYSV) have been reported in all the melon production areas of Xinjiang province and have been a serious problem for melon production [10]. Rapid detection and statistical analysis of virus diseases are very important for improving the quality of melon. Currently, methods including reverse transcription-polymerase chain reaction (RT-PCR), double antibody sandwich ELISA (DAS-ELISA) [12], protein A sandwich ELISA (PAS-ELISA), serological assay and antigen immune have been used to detect melon viruses [13,14,15]. The RT-PCR method has been widely used in the detection of WMV, CMV, and TMV, which has proven to be an attractive alternative for the identification of plant viruses [16,17,18]. However, to our knowledge, there are few reports on the simultaneous detection of the seven viruses (WMV, CMV, ZYMV, SqMV, TMV, PRSV, and MYSV) based on one-multiplex RT-PCR method in the field.

A RT-PCR detection system was developed to simultaneously detect seven viruses isolated from melon in Xinjiang province (WMV, CMV, ZYMV, SqMV, TMV, PRSV, and MYSV). RT-PCR technology with multiplex detection can provide an accurate, efficient, and fast method of detecting of melon viruses, providing a theoretical basis for timely prevention and treatment against viruses

## 2. Materials and Methods

### 2.1. Collection of Virus-Infected Leaves

**Figure 1:**
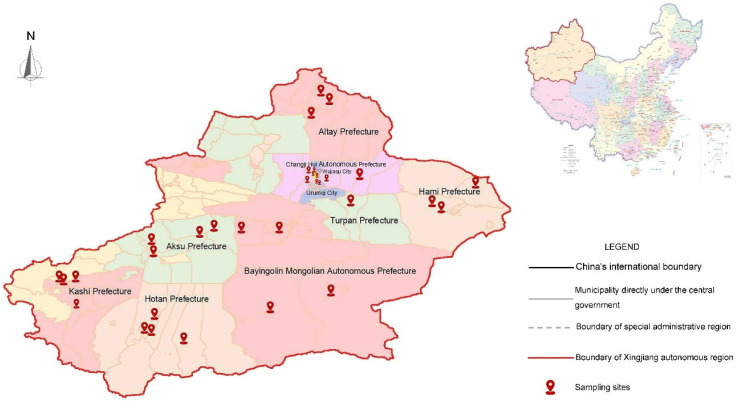
The map shows the locations of sampling sites across various prefectures in Xinjiang, marked by red pins. Prefectures include Aksu, Altay, Kashi, Hotan, Hami, Turpan, and Bayingolin Mongolian Autonomous Prefecture, with notable cities such as Urumqi and Changji Hui Autonomous Prefecture also highlighted. The boundaries of Xinjiang, China’s international borders, and various administrative regions are outlined, with a smaller inset map showing the location of Xinjiang within China.

**Figure 2:**
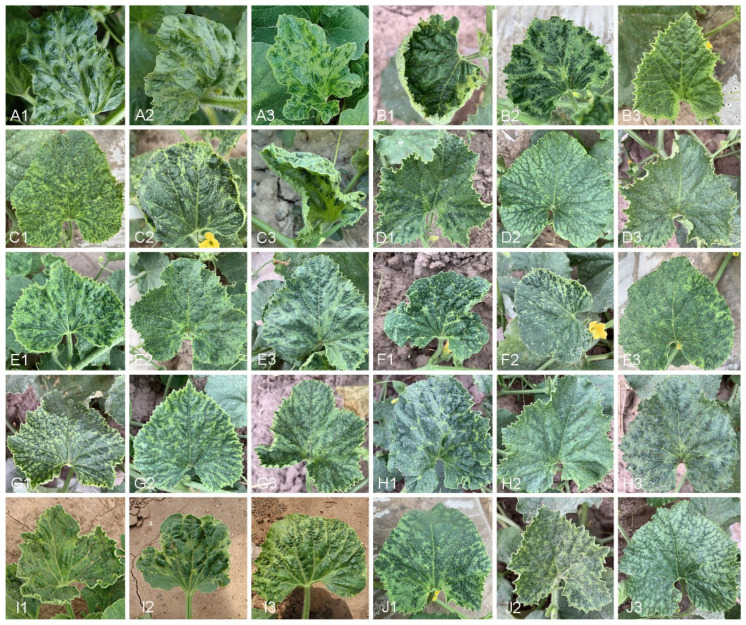
The images show different degrees of chlorosis, mosaic patterns, and leaf deformation in melon plants across treatments. Panels (**A1**–**J3**) depict variations in disease symptoms such as yellowing, curling, and blistering. Each row represents a different set of treatments, with individual images (**A1**–**J3**) highlighting specific responses of the leaves to potential stressors. These visible symptoms suggest the presence of viral or environmental stress, with severity and patterns differing across the treatments.

The fresh melon leaf samples were collected from healthy plants or plants infected with single or mixed destructive pathogens in the field of Xinjiang province (32 sampling sites in 10 prefectures) (Figure 1). A total of 242 samples were collected from different regions (Figure 1). These samples were stored in a liquid nitrogen container and transported to the laboratory for storage and further analysis (Figure 2).

### 2.2. Primer Design and Selection

The conserved fragments of coat protein (CP) genes of WMV, CMV, ZYMV, SqMV, TMV, PRSV, and MYSV were determined by alignment using DNAMAN. Based on the conserved regions, seven pairs of primers were designed using Primer software 5.0. All the primers were designed based on specificity and compatibility to facilitate reverse transcription and PCR. These primer sequences, expected size of amplification products, and the target genes are listed in Table 1.

### 2.3. Total RNA Extraction

Total RNA was extracted using the TRIZOL method [19] with slight modifications. About 2.0 mL of TRIZOL lysis buffer was added to the powdered melon leaf tissues and violently blended. Then, 600 μL of chloroform was added to the solution after a 4–5 min of incubation and vortexes to mix the solution. The supernatant was collected after centrifugation, and 0.6–7 times the volume of isopropanol was added and mixed by gently shaking the test tube. It was then horizontally placed to complete the reaction for 5 min. The solution was centrifugal, and the white precipitate was collected and rinsed with 75% alcohol two times. After 5 min, when the alcohol (attached on the tube wall) was naturally dried at room temperature, the precipitate was dissolved in 40 μL of DEPC-treated ddH_2_O. Total RNA was detected using agarose gel electrophoresis (10 g/L). The concentration and purity of total RNA was determined using UV spectrophotometry, and the solution was stored at −80 °C for further use.

### 2.4. RT-PCR Detection of Viruses

Using RevertAid^TM^ first-strand cDNA synthesis kits (Dingguo Changsheng Co., Ltd., Beijing, China), the first-strand cDNA of seven viruses were synthesized following the manufacturer’s instruction. A reaction mixture of 3 μg of total RNA, 0.6 μL of random hexamer primers, 0.4 μL of oligo (dT)18 primers, and nuclease-free ultrapure water to a total volume of 12 μL was added to a PCR tube (DEPC-treated). The reaction mixture was gently centrifuged, incubated at 70 °C for 5 min, cooled on ice immediately, and centrifuged. After centrifugation, about 1 μL of RiboLockTM RNase inhibitor (20 U/L), 4 μL of 5× reaction buffer, 1 μL of RevertAid^TM^ M-MLV reverse transcriptase (200 U/L), and 2 μL of dNTP Mix (10 mmol/L) were added to obtain a total volume of 20 μL. The solution was centrifuged after blending gently, then incubated at 42 °C for 60 min. The reaction was stopped by heating at 70 °C for 5 min, and the cDNA was stored at −80 °C.

The reaction mixture (25 μL) for singleplex RT-PCR consisted of 2.5 μL of 10× buffer (including 25 mmol/L MgCl_2_), 0.5 μL of dNTPs (10 mmol/L), 1 μL of each upstream and downstream primers (10 μmol/L), 0.3 μL of *Taq* DNA polymerase (2.5 U/μL), 1 μL of template cDNA, and 18.7 μL of ddH_2_O. The PCR conditions for the reaction were pre-denaturing at 94 °C for 4 min, followed by 35 cycles of denaturing at 94 °C for 40 s, annealing at varying temperatures (annealing temperature varied with primers) for 35 s, and extension at 72 °C for 60 s; the amplification was completed by holding the reaction mixture at 72 °C for 10 min. The product was stored at 4 °C. The PCR instrument was for the reaction, and the PCR product was visualized on agarose gel electrophoresis (10 g/L). Bio-Rad gel imaging system was used to observe the gel, and the results were recorded.

The PCR products were purified from the gels using the GenePure Gel Purification Kit (Shanghai Yubo Biotech Co., Ltd., Shanghai, China). Fragments were ligated into the pGEM-T Easy vector (Promega, Madison, WI, USA) and transformed into *Escherichia coli strain* JM109. Positive recombinant clones were selected and sequenced by Shanghai Yingjie (Biotech Co., Ltd., Shanghai, China). Three independent positive clones for each isolate were sequenced.

### 2.5. Development and Optimization of Multiplex PCR System

The positive recombinant clones of each virus identified by RT-PCR were mixed to use as a mixed-virus pool. Based on the single factor analysis method, the concentration of Mg^2+^, dNTPs, primer, *Taq* DNA polymerase, cDNA, and the annealing temperature were selected as the main factors and optimized. Each factor was set at seven levels in the optimized experiment. The concentrations of Mg^2+^ were 1.0, 1.5, 2.0, 2.5, 3.0, 3.5, and 4 mmol/L. The cDNA was 0.2, 0.4, 0.6, 0.8, 1.0, 1.2, and 1.4 μL. In the optimization experiment of the RT-PCR detection method, three treatments were designed for the total amount of primers, namely 2 × 10^−5^ μ mol, 5 × 10^−5^ µ mol, and 7 × 10^−5^ µ mol. In the experimental treatment with total primers amount of 2 × 10^−5^ μ mol, the molar ratios of primers for detecting WMV, CMV, ZYMV, SqMV, TMV, PRSV, and MYSV were set to 1:1:1:1:1, 2:2:2:1:1:1, and 10:9:8:5:3:3, respectively; in the experimental treatment with a total primers amount of 5 × 10^−5^ μ mol, the molar ratios of primers for detecting WMV, CMV, ZYMV, SqMV, TMV, PRSV, and MYSV were set to 1:1:1:1:1, 2:2:2:1:1:1, and 10:9:8:5:3:3, respectively; in the experimental treatment with total primers amount of 5 × 10^−5^ μ mol, the molar ratio of primers for detecting WMV, CMV, ZYMV, SqMV, TMV, PRSV, and MYSV was 1:1:1:1:1. The unit activities of *Taq* DNA polymerase were 0.25, 0.50, 0.75, 1.00, 1.25, 1.50, and 1.75 U; and the annealing temperatures were 50, 51, 52, 53, 54, 55, and 56 °C. In the gradient optimization of a single factor, the other factors were not changed. Each combination was repeated three times. The number of amplified bands was statistically calculated after multiplex RT-PCR. The best combination with high stability, clear and correct bands was selected as the multiplex RT-PCR reaction system.

### 2.6. Sensitivity and Specificity of the Multiplex RT-PCR

The sensitivity and specificity of this method were tested by positive recombinant clones. These positive recombinant clones were adjusted to the same initial concentration and diluted serially tenfold (10^5^ to 10^0^ copies/μL) with deionized water to serve as a template for testing the sensitivity of this multiplex RT-PCR.

The drop-out experiments were carried out to test the specificity of this multiplex RT-PCR, in which one pair was removed at a time to see whether the rest of the primers had cross-reacted [14]. Each virus-specific primer set was sequentially eliminated in this experiment.

The following formula was used to calculate the number of gene copies per microliter in each dilution: copies/μL = (6.02 × 10^23^) × (plasmid concentration [ng/μL] × 10^−9^)/(DNA length[bp] × 660) [18].

### 2.7. Detection of Field Samples Using the Multiplex RT-PCR

The established multiplex RT-PCR reaction system was used to detect the samples in the field. The detection results were compared with the single RT-PCR to verify the accuracy and stability of multiplex RT-PCR.

## 3. Results

### 3.1. Primer Specificity and Compatibility

**Figure 3:**
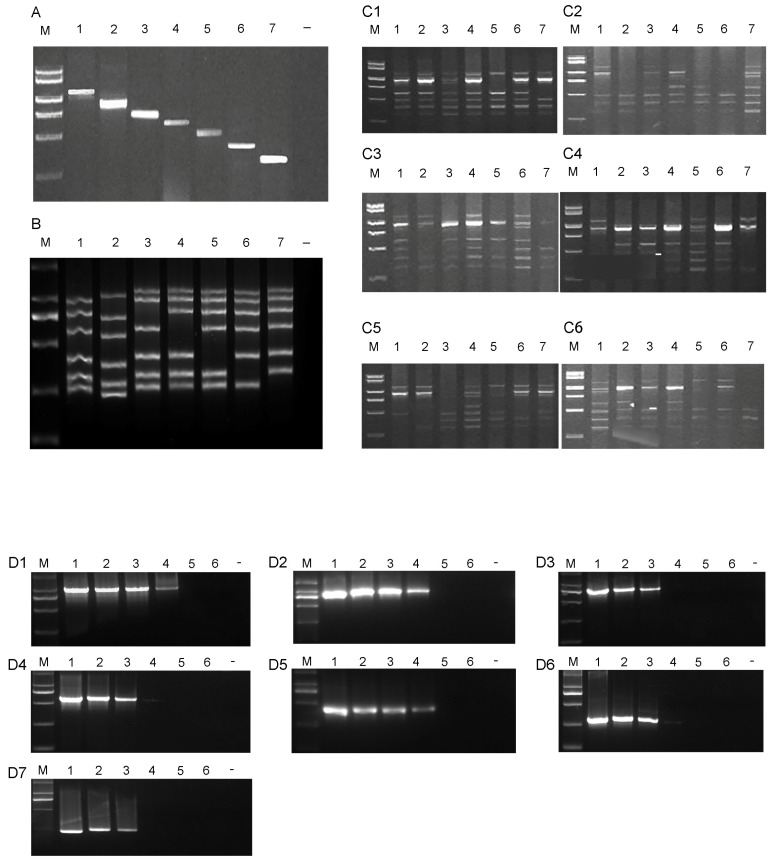
(**A**). The specificity analysis results of the multiplex RT-PCR detection method. The detection samples corresponding to lanes 1 to 7 only contain WMV, CMV, ZYMV, SqMV, TMV, PRSV, and MYSV, respectively. (**B**). The sensitivity tests of the multiplex RT-PCR detection method. The drop-out experiments were carried out to test the specificity of this multiplex RT-PCR, in which one pair was removed at a time to see whether the rest of the primers had cross-reacted.; lane 1: healthy plant (negative control); lane 2: detection of MYSV; lane 3: detection of PRSV; lane 4: detection of TMV; lane 5: detection of SqMV; lane 6: detection of ZYMV; lane 7: detection of CMV. (**C1**). Detection results of using different Mg^2+^ concentrations in multiplex RT-PCR amplification system. lane 1: at 1.0 mol/L; lane 2: at 1.5 mmol/L; lane 3: at 2.0 mmol/L; lane 4: at 2.5 mmol/L; lane 5: at 3.0 mmol/L; lane 6: at 3.5 mmol/L; lane 7: at 4.0 mmol/L. (**C2**). Detection results of using different template cDNA volumes in multiplex RT-PCR amplification system. lane 1: at 0.5 μL; lane 2: at 0.75 μL; lane 3: at 1 μL; lane 4: at 1.25 μL; lane 5: at 1.5 μL; lane 6: at 1.75 μL; lane 7: at 2 μL. (**C3**). Detection results of using different primer amounts in a multiplex RT-PCR amplification system; lane 1: at 2 × 10^−5^ μmol, and molar ratios of primers for WMV, CMV, ZYMV, SqMV, TMV, PRSV, and MYSV is 1:1:1:1:1:1:1; lane 2: at 2 × 10^−5^ μmol, and molar ratios of primers for WMV, CMV, ZYMV, SqMV, TMV, PRSV, and MYSV is 2:2:2:1:1:1:1; lane 3: at 2 × 10^−5^ μmol, and molar ratios of primers for WMV, CMV, ZYMV, SqMV, TMV, PRSV, and MYSV is 10:9:8:8:5:5:3:3; lane 4: at 5 × 10^−5^ μmol, and molar ratios of primers for WMV, CMV, ZYMV, SqMV, TMV, PRSV, and MYSV is 1:1:1:1:1:1:1; lane 5: at 5 × 10^−5^ μmol, and molar ratios of primers for WMV, CMV, ZYMV, SqMV, TMV, PRSV, and MYSV is 2:2:2:1:1:1:1; lane 6: at 5 × 10^−5^ μmol, and molar ratios of primers for WMV, CMV, ZYMV, SqMV, TMV, PRSV, and MYSV is 10:9:8:8:5:5:3:3; lane 7: at 7 × 10^−5^ μmol, and molar ratios of primers for WMV, CMV, ZYMV, SqMV, TMV, PRSV, and MYSV is 1:1:1:1:1:1:1. (**C4**). Detection results of using different dNTP concentrations in multiplex RT-PCR amplification system; lane 1: at 0.2 mmol/L; lane 2: at 0.4 mmol/L; lane 3: at 0.6 mmol/L; lane 4: at 0.8 mmol/L; lane 5: at 1.0 mmol/L; lane 6: at 1.2 mmol/L; lane 7: at 1.4 mmol/L; (**C5**). Detection results of different amounts of Taq DNA polymerase in multiplex RT-PCR amplification system; lane 1: at 0.25 U; lane 2: at 0.5 U; lane 3: at 0.75 U; lane 4: at 1.0 U; lane 5: at 1.25 U; lane 6: at 1.5 U; lane 7: at 1.75 U. (**C6**). Detection results of using different annealing temperatures in multiplex RT-PCR method; lane 1: at 50 °C; lane 2: at 51 °C; lane 3: at 52 °C; lane 4: at 53 °C; lane 5: at 54 °C; lane 6: at 55 °C; lane 7: at 56 °C. (**D**). The detection limits of the multiplex RT-PCR assays. The detection limits of this multiplex PCR were conducted by a series of sensitivity tests. The positive clone vector was adjusted to the same initial concentration and diluted serially ten-fold (10^5^ to 10^10^ copies/μL) to serve as a template in the optimized multiplex PCR. (**D1**). The detection limits of WMV; 1–6 stand for 10^5^ to 10^0^ copies/μL. (**D2**). The detection limits of CMV;1–6 stand for 10^5^ to 10^0^ copies/μL. (**D3**). The detection limits of ZYMV; 1–6 stand for 10^5^ to 10^0^ copies/μL (**D4**). The detection limits of SqMV; 1–6 stand for 10^5^ to 10^0^ copies/μL. (**D5**). The detection limits of TMV; 1–6 stand for 10^5^ to 10^0^ copies/μL. (**D6**). The detection limits of PRSV; 1–6 stand for 10^5^ to 10^0^ copies/μL. (**D7**). The detection limits of MYSV; 1–6 stand for 10^5^ to 10^0^ copies/μL. ”–“represents deionized water as control. M: DNA marker (100 bp–2000 bp).

The primer specificity of the multiplex RT-PCR assay was initially optimized by amplifying a specific virus. The seven primer sets were added into multiplex RT-PCR systems and to amplify the specific virus. These PCR productions were signal and bright band, which confirmed the specificity of the primer (Figure 3A). The drop-out experiments also confirmed the specificity of this multiplex RT-PCR (Figure 3B).

To further determine the specificity of the multiplex RT-PCR assays, the PCR products were cloned and sequenced. The sequence results showed that all the amplifications were specific to the corresponding virus, which reconfirmed the specificity and accuracy of the method.

### 3.2. The Optimized Multiplex RT-PCR

The main six factors (Mg^2+^ concentration, template cDNA, primer proportion, dNTP concentration, unit activity of Taq DNA polymerase, and annealing temperature) of multiplex RT-PCR were optimized in this study. An optimum multiplex RT-PCR reaction system (25 μL) was developed based on a combination of different concentrations for analysis of each factor in RT-PCR (Figure 3(C1–C6)). The best combination of each factor was presented as follows: Mg^2+^ concentration, 2.0 mmol/L; template cDNA, 2 μL, primer proportion (total mol 2 × 10^−5^ μmoL), molar ratios 10:9:8:8:5:5:3:3; dNTPs concentration, 1 mmol/L; unit activity of Taq DNA polymerase, 1 U; annealing temperature, 50 °C.

### 3.3. Detection Limits of Multiplex PCR

The detection limits of this multiplex PCR were conducted by a series of sensitivity tests. The positive clone vector was adjusted to the same initial concentration and diluted serially ten-fold (10^5^ to 10^10^ copies/μL) to serve as a template in the optimized multiplex PCR. For different viruses, the difference in the detection limit was found in the multiplex PCR. For ZYMV and MYSV, the detection limits were 10^3^ copies/μL. While for WMV, SqMV, PRSV, CMV, and TMV, the detection limits were 10^2^ copies/μL (Figure 3(D1–D7)).

### 3.4. The Application of Multiplex RT-PCR in the Field

**Figure 4:**
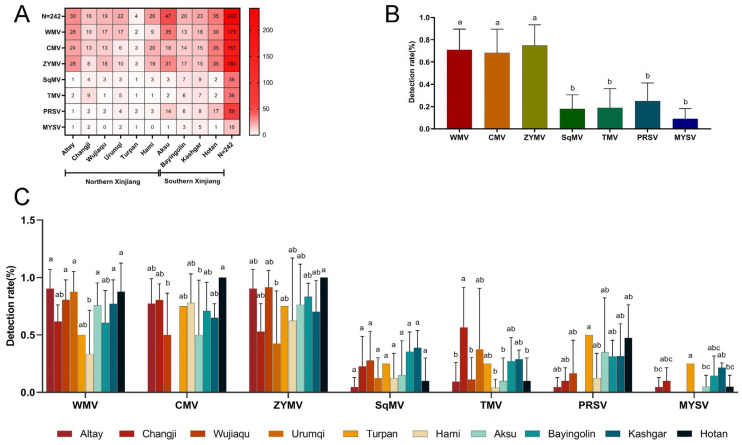
(**A**). The heatmap displays the distribution and frequency of various viral diseases affecting melon crops across different regions in northern and southern Xinjiang. The regions include Altay, Changji, Wujiaqu, Urumqi, Turpan, Hami, Aksu, Bayingolin, Kashgar, and Hotan. Viral diseases represented are *Watermelon Mosaic Virus* (WMV), *Cucumber Mosaic Virus* (CMV), *Zucchini Yellow Mosaic Virus* (ZYMV), *Squash Mosaic Virus* (SqMV), *Tobacco Mosaic Virus* (TMV), *Papaya Ringspot Virus* (PRSV), and *Melon Yellow Spot Virus* (MYSV). The color intensity corresponds to the number of cases, with red indicating higher incidence. The total number of cases (N = 242) per virus and per region is shown, with regions in southern Xinjiang showing a generally higher disease incidence compared to northern Xinjiang. (**B**). This figure shows the results of differential analysis of detection rates among different viruses in the Xinjiang region. The data of each virus detection rate on the horizontal axis is the average of the virus detection rates in 10 locations in Xinjiang. This figure shows the analysis results of the differences in virus detection rates between different regions. The horizontal axis represents different virus types, and the vertical axis represents virus detection rates. (**C**). Detection rate of *Watermelon Mosaic Virus* (WMV), *Cucumber Mosaic Virus* (CMV), *Zucchini Yellow Mosaic Virus* (ZYMV), *Squash Mosaic Virus* (SqMV), *Tobacco Mosaic Virus* (TMV), *Papaya Ringspot Virus* (PRSV), and *Melon Yellow Spot Virus* (MYSV) at different locations of Xinjiang. The reaction experimental results of lowercase letters reach the 0.05 significance level. Different letters represent significant differences between groups, and the same letters or shared letters represent insignificant differences between groups.

The optimized multiplex RT-PCR was used for clinical detection of the samples from the field. Among the 242 samples, all samples were infected by one or more than one virus. ZYMV may be the most severe virus affecting melons in Xinjiang province, as 184 samples were infected by ZYMV (Figure 4). WMV and CMV were the more serious viruses, since 179 and 161 samples were infected with WMV and CMV, respectively (Figure 4A). There were 16 samples infected by MYSV, which confirmed MYSV was the minimum threat to melon production (Figure 4A). The detection rates of WMV, ZYMV, and CMV are significantly higher than other viruses (Figure 4B), indicating that these three viruses are currently the dominant species of melon viruses in Xinjiang.

The distribution and incidence rates of these Xinjiang melon regions were statistically recorded. The results showed that the incidence status of seven viruses in northern Xinjiang regions was greater than that in southern regions (Figure 4A). The mixed infections among the virus in northern Xinjiang regions were more complex than in southern regions. The detection rates of each virus vary in different regions, but overall, the detection rates of WMV, ZYMV, and CMV in most areas are higher than the other four viruses. (Figure 4C).

The infection manners of these viruses were analyzed in the field. Among 242 samples, 100 samples were infected by more than 1 virus. A total of 45 samples were mixed infected by 3 viruses, of which WMV, ZYMV, and CMV mixed infected was most common in the field. A total of 21 samples were mixed infected by 4 viruses, and 7 samples were by 5 viruses. There were 3 samples mixed infected by 7 viruses.

## 4. Discussion

**Figure 5:**
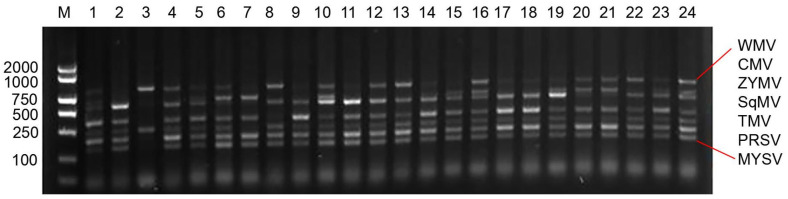
Results of multiplex RT-PCR detection method for virus disease samples from different regions; M: Marker; 1–24: Samples of melon plants infected by virus diseases in different regions of Xinjiang.

The multiplex RT-PCR was developed, optimized, and applied to the detection of seven viruses. Multiplex RT-PCR has been widely used for simultaneous detection of pathogenic microorganism, which decreases the risk of contamination, saves time, and reduces the cost [14]. One multiplex RT-PCR method was developed by optimizing the main four factors (Mg^2+^ concentration, primer proportion, unit activity of Taq DNA polymerase, and annealing temperature) (Figure 5). The specificity and detection limits were further tested to use in the field. The field experiment showed that this method had high accuracy, fast liquidity, and high flux [20], and can simultaneously detect a variety of melon virus diseases in the field, which may have been widely applied in food quality inspection, pathogen identification, species attribution, and positive screening [4,8].

Many factors may influence the multiplex RT-PCR, including primer design, template quality, reaction conditions, and the concentrations of reagents like dNTPs, MgCl_2_, and Taq polymerase. Previous studies suggested that these factors which influenced RT-PCR also had a significant impact on multiplex RT-PCR [20]. DNA *Taq* polymerase, the core factor affecting the specificity of PCR amplification, has always been the main optimizing factor in multiplex RT-PCR systems. The optimizing test of unit activity of DNA *Taq* polymerase in this study suggested that multiplex RT-PCR amplification was the best when DNA *Taq* polymerase was 0.75 U (Figure 3). Mg^2+^, as an activator of DNA *Taq* polymerase, plays an important role in the PCR assay, which can regulate the activity of DNA *Taq* polymerase, thereby affecting the specificity of PCR amplification. Studies have shown that a deficiency or an excess of Mg^2+^ causes a significant impact on the specificity of PCR. In this study, when the Mg^2+^ concentration was higher than 3.0 mmol/L, amplification was inhibited, performing incomplete and fuzzy band amplification; when the concentration was lower than 3.0 mmol/L, the activity of DNA *Taq* polymerase was inhibited, and the PCR assay could only amplify partial fragments but not all target products. Meanwhile, the effects of annealing temperature should be taken into account in multiplex RT-PCR systems. A low annealing temperature (50 °C) was used in this multiplex RT-PCR after comprehensive analysis of the GC content and combination ability of the primers. To guarantee stability and accuracy of subsequent detection, several important factors should be optimized and screened to develop multiplex RT-PCR systems.

The detection results suggested that the incidence rate of virus diseases in the northern Xinjiang region was higher than that in the southern region. The same virus presented varying incidence rates in different regions. The modest temperature and appropriate humidity in the northern Xinjiang region provide favorable conditions for virus spread. Viruses mainly spread through three pathways: sap contact, seed, and media [21]. Furthermore, there are far more greenhouses in the northern Xinjiang region than in southern Xinjiang, especially in Changji and Nongliushi 102 missions, the greenhouses provide wintering sites for the origin of viruses and transmission media. Effective measures should be taken to avoid further spread of virus diseases in these areas.

## Figures and Tables

**Table 1:** The primers of the multiplex RT-PCR.

Primer Name	Nucleotide Sequence (5′-3′)	Product Size (bp)
WMV1-F	GAGGAACTGTGGTTCATGTC	1149
WMV1-R	GCAGTGTGCCTCTCAGTATT	
CMV1-F	GACAGTTGGGAATCGGA	980
CMV1-R	AACAGGGAGCAAGAGGA	
ZYMV-F	CACGAAGGACAAGGATGTGA	789
ZYMV-R	ACATTGCTAAGAGCTGCTGC	
SqMV1-F	GGATGCCTTTGGCTATTGG	604
SqMV1-R	GCCTCCTCGTGGCTTTGTA	
TMV-F	TTTTGGAGGAATGAGTTT	397
TMV-R	AGGGAAAAACACTATGC	
PRSV-F	CTCGTGCCACTCAATCTCAA	300
PRSV-R	TTCCACTGTGTGTCTCTCCG	
MYSV-F	TTGAGGCAGGATCTGAAGTC	250
MYSV-R	GGCCAATCTGATCCAGAGTA	

## Data Availability

The original contributions presented in the study are included in the article, further inquiries can be directed to the corresponding authors.
